# The Asian Lineage of Zika Virus: Transmission and Evolution in Asia and the Americas

**DOI:** 10.1007/s12250-018-0078-2

**Published:** 2019-01-25

**Authors:** Tao Hu, Juan Li, Michael J. Carr, Sebastián Duchêne, Weifeng Shi

**Affiliations:** 1 Key Laboratory of Etiology and Epidemiology of Emerging Infectious Diseases in Universities of Shandong, Taishan Medical College, Taian, 271000 China; 2 Global Station for Zoonosis Control, Global Institution for Collaborative Research and Education (GI-CoRE), Hokkaido University, Sapporo, 001-0020 Japan; 3 National Virus Reference Laboratory, School of Medicine, University College Dublin, Belfield, Dublin 4, Ireland; 4 Department of Biochemistry and Molecular Biology, Bio21 Molecular Science and Biotechnology Institute, University of Melbourne, Parkville, VIC 3020 Australia

**Keywords:** Zika virus (ZIKV), Asian lineage, Transmission, Evolution, *R*_*e*_

## Abstract

Since first isolation in 1947 from the Zika forest in Uganda, Zika virus (ZIKV) has been principally known as a benign agent associated with sporadic human infections in a restricted number of African countries. However, during 2015–2016, an Asian lineage of ZIKV caused an unprecedentedly large outbreak in the Americas and sizeable numbers of exported cases across the globe. In this review, we critically appraise the recent advances in molecular epidemiological studies of ZIKV performed to date, and we highlight the pivotal role played by genomic surveillance in elucidating the origins, dissemination and evolution of the Asian lineage of ZIKV in Asia and in the Americas.

Zika virus (ZIKV), a mosquito-borne flavivirus, has been known for over 70 years. The positive sense, single-stranded RNA genomic structure of ZIKV and processing of the single encoded large polyprotein is characteristic of other flaviviruses, such as yellow fever virus and dengue virus. The majority of ZIKV infections are asymptomatic; however, in some cases can have a febrile presentation with mild clinical symptoms, such as, headache, skin rashes and conjunctivitis, which are typically self-limiting, resolve without consequence and do not require anti-viral treatment (Calvet *et al.*^2016^; Hamel *et al.*^2016^; Song *et al.*^2017^). Notably, prior to 2007, a distinguishing feature from other closely related flaviviruses able to cause large scale outbreaks, was that ZIKV had been only reported to be associated with sporadic human infections in a small number of African countries (Calvet *et al.*^2016^; Hamel *et al.*^2016^; Song *et al.*^2017^). In 2007, for the first time, ZIKV was linked to a large-scale epidemic on Yap Island in Micronesia, with a total of at least 49 laboratory-confirmed cases (Lanciotti *et al.*^2008^; Duffy *et al.*^2009^). ZIKV then caused an even larger outbreak in French Polynesia and surrounding islands in 2013 (Cao-Lormeau *et al.*^2014^; Hancock *et al.*^2014^; Ioos *et al.*^2014^). It was in this outbreak that ZIKV was first linked to Guillain-Barré syndrome (GBS), a severe autoimmune neuropathology (Oehler *et al.*^2014^; Cao-Lormeau *et al.*^2016^). Surprisingly, ZIKV subsequently disseminated across the Americas, where it caused an outbreak on an unprecedented scale for this previously considered benign agent. By the end of 2016, at least 48 countries and territories in the Americas had reported more than 175,000 confirmed ZIKV cases (Ikejezie *et al.*^2017^). A retrospective investigation has revealed that ZIKV-associated microcephaly was first reported in French Polynesia during the 2013–2014 epidemic (Besnard *et al.*^2016^). More importantly, ZIKV was first definitively associated with microcephaly in the South American outbreak: a condition where a neonate has a significantly smaller head circumference than normal, or the cranium ceases normal development after birth (Broutet *et al.*^2016^; Driggers *et al.*^2016^; Mlakar *et al.*^2016^). By February 2017, a total of 2654 cases of ZIKV-related fetal microcephaly and central nervous system (CNS) malfunction were reported to the World Health Organization, with approximately 90% of the cases distributed in Brazil (https://www.who.int/emergencies/zika-virus/situation-report/2-february-2017/en/).

In the preceding 3 years, there have been numerous studies describing the molecular epidemiologic and genomic characterization of ZIKV identified from the 2015–2016 outbreak. The first important study that examined the ZIKV-associated American outbreak was published in *Science* where Faria and colleagues dated the transmission to have occurred via a single introduction event from Pacific Islands between May and December of 2013 (Faria *et al.*^2016^). Three seminal studies were then subsequently published in the same issue of *Nature* in June of 2017 which delineated the transmission and evolution of ZIKV across the Americas (Faria *et al.*^2017^; Grubaugh *et al.*^2017^; Metsky *et al.*^2017^; Worobey 2017). Faria *et al.* revealed that Bahia province in northeastern Brazil was the epicenter of the outbreak in the Americas, where ZIKV first became established and then subsequently disseminated nationally and internationally (Faria *et al.*^2017^). Metsky and colleagues determined when the virus was transmitted to Central America and the Caribbean islands (Metsky *et al.*^2017^). Grubaugh and co-workers further analyzed when and how often the virus was disseminated into the USA and pointed out that ZIKV had most likely been imported multiple times from the Caribbean (Grubaugh *et al.*^2017^). Subsequently, Thézé and colleagues reported that ZIKV was imported into Central America and Mexico via multiple independent introduction events. In particular, one introduction, likely from Brazil via Honduras, led to most infections in Central America and Mexico from late 2014 onwards (Thézé *et al.*^2018^). In this mini-review, we critically appraise the current state of knowledge with regards to the transmission and evolution of the Asian lineage of ZIKV in the unprecedentedly large epidemic in the Americas and highlight the role of Southeast Asia in the evolution of this agent.

## Transmission and Evolution of the Asian Lineage of ZIKV

Previous studies have shown that ZIKV has diverged into two principal genetic lineages: African and Asian (Faria *et al.*^2016^; Pettersson *et al.*^2016^; Zhang *et al.*^2016^). Some researchers proposed a three-lineage classification nomenclature where the Asian lineage was further divided into two separable sub-lineages (Gubler *et al.*^2017^) or, alternatively, the African strains were further divided into two sub-lineages based on phylogenetic analysis of partial genomic sequences (Shen *et al.*^2016^). The later study also reported an African sister group to all currently known ZIKV major lineages (Shen *et al.*^2016^) which suggested that the Asian lineage originated from the African lineage (Gong *et al.*^2016^). This interesting finding clearly warrants further investigation and more comprehensive sampling and full genome sequencing.

**Fig. 1:**
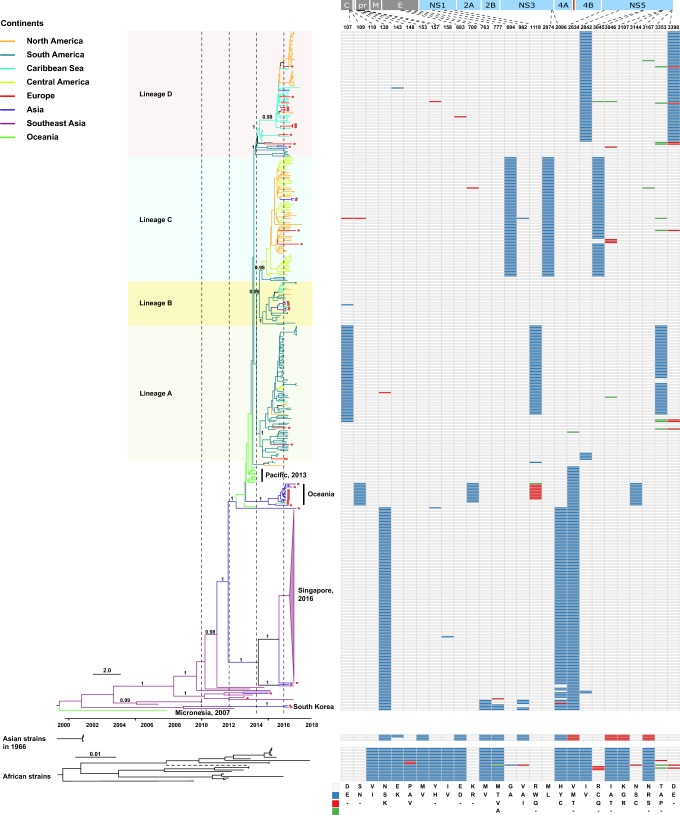
Phylogeny and molecular characterization of the Asian lineage of ZIKV. Full-length ZIKV genomes (n = 448) derived from the Asian lineage were assembled and a phylogenetic analysis using BEAST 1.8.4 was performed (Drummond *et al.*^2012^). The GTR + Γ nucleotide model was applied to account for rate variation among sites, with four categories for the Γ distribution. A log-normal distributed relaxed molecular clock was employed and the Bayesian skyline coalescent was designated as the tree prior. A Markov chain Monte Carlo with one hundred million steps was run twice independently and the first 10% were removed as burn-in. The posterior probability distribution of the parameters and trees from the two independent runs was combined using LogCombiner embedded in BEAST 1.8.4. Viruses identified from the ZIKV African lineage and from Indonesia cases in 1966 were also downloaded and phylogenetically analyzed using Maximum Likelihood. In the African lineage, one strain, ArD142623 (GenBank No. KF3838120), had a long branch length, which is displayed using a dashed line. In the Asian lineage, tips with circles represent confirmed ZIKV imported cases. Line color represents the collection site of the virus and the classification of the countries into different regions was based on Wikipedia (https://www.wikipedia.org/). In the right panel, different colors represent different amino acids, which have been shown in the legend in the bottom right of the panel.

The prototypic strain of the ZIKV Asian lineage was first isolated in 1966 from mosquito pools collected in Bentong, Malaysia (Marchette *et al.*^1969^). Although serological studies revealed that the first documented ZIKV outbreak in Asia may have occurred in central Java, Indonesia in 1977 (Olson *et al.*^1981^) and ZIKV infections are possibly more widespread and may be circulating asymptomatically in Southeast and South Asia (Musso and Gubler 2016), only very limited partial genomic sequences are available. However, in 2007, the first documented large-scale outbreak of ZIKV was reported on a remote Pacific island, Yap in Micronesia (Lanciotti *et al.*^2008^; Duffy *et al.*^2009^). Complete genome sequencing showed, conclusively, that the outbreak arose from the introduction of a ZIKV belonging to the Asian lineage (Lanciotti *et al.*^2008^). During 2013–2014, the virus caused outbreaks in four other Pacific islands with the outbreak in French Polynesia resulting in thousands of suspected human infections (Cao-Lormeau *et al.*^2014^; Hancock *et al.*^2014^; Ioos *et al.*^2014^). Phylogenetic analysis revealed that the ZIKV identified from laboratory-confirmed cases during this outbreak also belonged to the Asian lineage, which potentially originated in Southeast Asia (Cao-Lormeau *et al.*^2014^). However, these two outbreaks in the Pacific likely may have arisen from two independent introductions events from Southeast Asia (Delatorre *et al.*^2018^), or another unsampled Asian location, because they clustered together with strains from Southeast Asia in different positions of the tree (Fig. 1). Later in 2016, Chinese travelers returning from Fiji and Samoa were confirmed to be infected by ZIKV (Zhang *et al.*^2016^). Genomic sequencing showed that these ZIKVs did not cluster with those responsible for the outbreak in the Americas (Shi *et al.*^2016^). Alternatively, they formed a separate cluster outside the ZIKV Asian lineage in the Americas (Shi *et al.*^2016^), and strains of these Oceanian cases likely represent ZIKVs circulating locally within the Pacific which diverged from those responsible for the 2013–2014 outbreak.

It is noteworthy that there were several ZIKVs identified from Haiti during an outbreak of chikungunya fever in the Caribbean in mid-2014 (e.g. GenBank Accession Nos. KY415986–KY415991), which also segregated within the American ZIKV cases (Lednicky *et al.*^2016^). However, the first confirmed ZIKV infection was reported in northeastern Brazil in February of 2015, the presumed epicenter of the American outbreak (Faria *et al.*^2016^, ^2017^). From the tree, we can see that shortly after ZIKV was imported into the Americas, it diverged into four major sub-lineages in a relatively short period of time between late 2013 and early 2014, probably in Brazil as strains from Brazil were always located basally (Fig. 1). The majority of viruses within sub-lineage A were from South America. Sub-lineage B was also mainly from South America; however, some viruses were transmitted to the Caribbean regions. Most of the viruses in sub-lineage C were from both Central and North America, and, finally, sub-lineage D was populated with viruses from the Caribbean and North America. Therefore, although dissemination of the virus from Brazil to Central and North America, and to the Caribbean occurred independently many times (Fig. 1), the predominant ZIKVs circulating in Central and North America and the Caribbean appear to have been transmitted via a small number of dominant importation events.

**Fig. 2:**
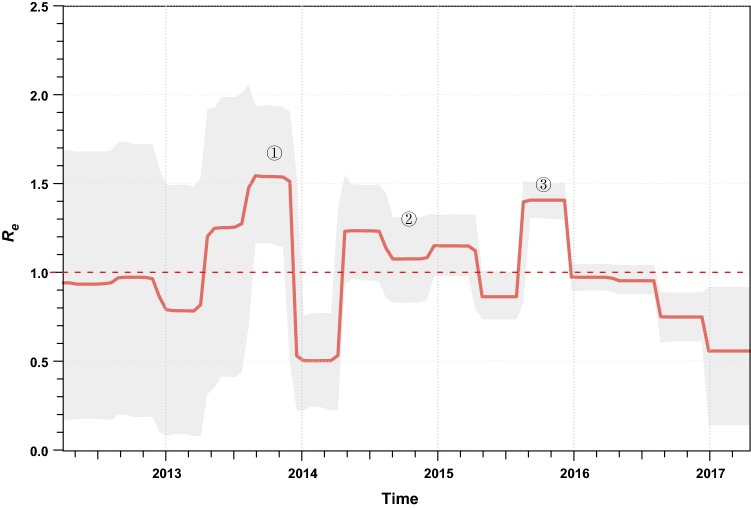
Effective reproductive number (*R*_*e*_) of ZIKV of the Asian lineage through time. The effective reproductive number (*R*_*e*_) was estimated using a birth–death skyline approach implemented in BEAST 2 with ZIKVs identified since 2013. An uncorrelated log-normal relaxed clock model and the GTR + Γ nucleotide substitution model were used. A Markov chain Monte Carlo was run with one hundred million steps and the first 10% were removed as burn-in. In the serially sampled birth–death skyline model, the priors for the molecular clock rate, *R*_*e*_, the sampling proportion, and the rate at which patients recover were set according to a previous study, which were also supported by an alternative approach allowing for sampling heterogeneity (data not shown) (Thézé *et al.*^2018^). The red line and lighter shading represented the median posterior estimate of *R*_*e*_ and its 95% highest posterior density credible intervals, respectively. This figure was created using the R-package bdskytools (available at https://github.com/laduplessis/bdskytools).

We estimated the effective reproductive number (*R*_*e*_; i.e. the average number of secondary infections) of ZIKV over time using a birth–death skyline approach (Stadler *et al.*^2013^) implemented in BEAST 2 (Bouckaert *et al.*^2014^). There were three major phases in which ZIKV rapidly spread with *Re* > 1 (Fig. 2). The first phase was estimated to have occurred between March and November of 2013 when the virus caused the large-scale outbreaks on the Pacific islands (Fig. 2). Interestingly, this is also the estimated time when ZIKV was introduced into South America (Faria *et al.*^2016^) and the time when the major South American sub-lineages diverged (Fig. 1). Therefore, the rapid increase in the average number of transmissions in the Pacific might have facilitated, or even been a prerequisite for this virus transmission event. The second phase where ZIKV population size expanded was between March 2014 and March 2015 (Fig. 2). After the virus was imported into South America, it evolved under an *R*_*e*_ > 1 for approximately one year; then, towards the end of this phase, the first confirmed ZIKV infection case was reported in northeastern Brazil (Faria *et al.*^2016^, ^2017^). More importantly, it is during this phase that many early virus dissemination events from Brazil to Central America and the Caribbean might have occurred, although evidence is scant due to undersampling (Faria *et al.*^2017^; Metsky *et al.*^2017^). Following phase 2, there was a short period of decrease in *R*_*e*_. We speculate that this might be attributable to decreases in population numbers of competent mosquito vectors for ZIKV transmission, as this time period overlaps with the lowest annual ambient temperatures in the southern hemisphere winter. However, even during this phase, ZIKV may have also circulated continuously and undetected in tropical Central America and the Caribbean (Thézé *et al.*^2018^). The third phase began from approximately August of 2015 and ended in late 2015 (Fig. 2). During this phase, the virus continued to circulate in the Americas. It is thought that after this phase the virus was imported into the United States and led to the first autochthonous transmission events in Florida (Grubaugh *et al.*^2017^). ZIKV laboratory-confirmed events and the cases of congenital malformations subsequently decreased in South America in 2016 and may, at least be partly attributable to the decrease in the number of susceptible individuals to maintain chains of transmission after such an unprecedently large outbreak; however, the proximate and ultimate causes are likely complex and clearly warrant further investigation.

## Contemporary ZIKV Outbreaks in Southeast Asia

Between 2014 and 2016, ZIKV outbreaks and sporadic autochthonous transmission events were also reported in several Southeast and South Asian countries, including Bangladesh, Cambodia, India, Indonesia, Lao People’s Democratic Republic, Malaysia, Maldives, Myanmar, Philippines, Singapore, Thailand and Vietnam (Lim *et al.*^2017^), suggesting the widespread circulation of ZIKV in Southeast and South Asia. Of particular note is the ZIKV outbreak in Singapore starting from August of 2016, with a total of 455 reported cases between August 27 and November 30, 2016 (Singapore Zika Study 2017). Phylogenetic analysis showed that ZIKV strains identified in the Singaporean outbreak were not descended from those circulating in the Americas. Alternatively, they were suggested to diverge from ZIKVs circulating locally in Southeast Asia (Singapore Zika Study 2017). In addition, Thailand reported the largest number of confirmed ZIKV cases (~ 700) in Asia and more than 200 ZIKV cases were reported in Vietnam in 2016 (Lim *et al.*^2017^). Apart from these three countries with hundreds of ZIKV cases, few sporadic autochthonous infections were reported in the remaining countries mentioned above. In particular, though far fewer in number, at least three suspected microcephaly cases (two from Thailand and one from Vietnam) were reported (Lim *et al.*^2017^; Moi *et al.*^2017^; Wongsurawat *et al.*^2018^). Precisely why congenital malformations appear more frequently in the South American cases than Asian cases is unclear but may be attributable to host genetic factors conferring differing levels of susceptibility to infection (Bayer *et al.*^2016^; Jagger *et al.*^2017^; Kenney *et al.*^2017^; O’Brien *et al.*^2014^). Genome wide association studies could potentially be employed to identify single nucleotide polymorphisms responsible for this phenomenon. Viral genomic sequencing revealed that ZIKV from the two microcephaly cases from Thailand belonged to the Asian lineage but did not cluster with the ZIKVs responsible for the outbreak in the Americas (Wongsurawat *et al.*^2018^). Therefore, virus-specific adaptations may also play a role in the differing pathogenesis which we explore in more detail below.

## Imported ZIKV Cases Outside the Americas

The unprecedentedly large 2015–2016 ZIKV outbreak was mainly centered upon the Americas. Contemporaneously, however, there were also thousands of imported ZIKV cases outside the Americas. For example, more than two thousand ZIKV cases were imported into Europe by travelers returning from ZIKV-endemic regions (Wilder-Smith *et al.*^2018^). Hypothetically, the number of imported ZIKV cases in Europe and other regions may be greatly underestimated due to a number of factors. These include the high percentage of sub-clinical infections, the overlapping clinical presentation of Zika with numerous acute-onset, travel-associated, influenza-like illnesses, the narrow window and low titer viremia precluding definitive molecular confirmation by amplification-based detection approaches, and, finally, the paucity of flavivirus-specific serological assays allowing the unambiguous discrimination from other related agents (Lim *et al.*^2017^). Notably, many highly populous countries in Asia have also reported imported ZIKV cases, including China, India and Japan (Lim *et al.*^2017^), and greater than one billion Asian people live in areas with competent mosquito vectors and are therefore at high risk for large Zika outbreaks (Lim *et al.*^2017^; Siraj and Perkins 2017). Hypothetically, certain human populations may be less susceptible to ZIKV and the virus may circulate silently without detection in large population centers further adapting to more effectively transmit among humans and, thus, represent reservoirs for the origin of the founder viruses in large-scale outbreaks in immunologically naïve populations elsewhere with greater numbers of susceptible individuals. Also, whether the hyperendemicity of the four serologically distinct serotypes of dengue viruses in Southeast Asian countries impacts upon the transmission of ZIKV is unclear but may conceivably confer differing susceptibility to ZIKV which warrants further study. It should be noted that apart from the Americas, Southeast Asia has also been found, on multiple occasions, to export ZIKV to the Pacific islands and East Asia. Phylogenetic analysis revealed that these imported ZIKVs scattered across the Asian lineage, without clear geographic clustering (Fig. 1). However, the long-distance dissemination of the virus should be given special attention as evidenced by the finding that the progenitor strains responsible for the American ZIKV outbreak were likely transmitted from the Pacific via a single importation event (Faria *et al.*^2016^).

## Molecular Characterization of Asian Lineage ZIKV

It has been controversial whether the Asian or African lineages of ZIKV are intrinsically more virulent (Rossi *et al.*^2018^), although strains of the Asian lineage are responsible for all known ZIKV outbreaks. However, different researchers have shown that there were distinct amino acid differences between ZIKV strains from the African and Asian lineages (Fig. 1) (Pettersson *et al.*^2016^; Ye *et al.*^2016^) and, also, between strains isolated from humans and mosquitoes (Wang *et al.*^2016^), some of which have been experimentally verified to have important roles in ZIKV transmission and pathogenesis. For example, the S139N substitution in the prM protein has been found to significantly increase the neurovirulence of ZIKV, which might, as a consequence, have caused an excess of microcephaly cases and other congenital CNS malformations during the American outbreak (Yuan *et al.*^2017^). In addition, the A982V substitution in the NS1 protein was reported to increase NS1 antigenemia, which was suggested to have enhanced the ZIKV infectivity and prevalence in mosquitoes and may have contributed to the widespread transmission of ZIKV in the Americas (Liu *et al.*^2017^). The evolutionary pattern of these biologically critical amino acid positions in ZIKV has been illustrated elsewhere (Liu *et al.*^2019^).

Apart from the previously reported substitutions that distinguish the two main ZIKV lineages, we have also identified a panel of sub-lineage-specific amino acid substitutions (Fig. 2), including residues 109, 709, 1118 and 3144 in the Oceanian origin cases imported to China from returning travelers, and 107, 1118 and 3353 in sub-lineage A, 894, 2074 and 3045 in sub-lineage C, and 2842 and 3398 in sub-lineage D. These sub-lineage-specific amino acid substitutions may be without phenotypic consequence and thus have a neutral effect on viral fitness if they arise from a founder effect; however, they represent the further diversifying evolution of ZIKV in the Asian lineage which may contribute to a differing propensity for transmission and/or pathogenesis.

In summary, although the ZIKV outbreak in the Americas ended suddenly (Netto *et al.*^2017^), just like its unexpected beginning, genomic sequencing has elucidated the origins, transmission, evolution and factors underlying the differing pathogenic properties of ZIKV in the Americas and in Southeast Asia. Although recent advances have revealed the potential biological significance of several specific amino acid substitutions, these mutations should be further verified employing animal models and the biological functions of other Asian lineage-specific amino acid substitutions should also be explored. Considering the extensively distributed nature of mosquito vector species which are competent for ZIKV transmission across Southeast Asia, India and southern China, previous outbreaks caused by ZIKV and other related flaviviruses, the long-term (likely asymptomatic) circulation and adaptation to humans, the underestimated genetic diversity of ZIKV in this region due to inadequate sampling, and the ongoing ZIKV epidemic in India (http://www.promedmail.org/direct.php?id=20181004.6069063) and the recent Chinese imported case from the Maldives, a comprehensive survey of ZIKV and other flaviviruses in competent vectors in the Eastern Pacific region is urgently required. This would allow a finer scale delineation of the extant genetic diversity and detailed pathogenesis studies and, critically, to facilitate a rational, evidence-based development of vaccines to stable epitope targets to mitigate further disease outbreaks.
